# Methylstat sensitizes ovarian cancer cells to PARP-inhibition by targeting the histone demethylases JMJD1B/C

**DOI:** 10.1038/s41417-025-00874-z

**Published:** 2025-02-06

**Authors:** Franziska Maria Schwarz, Daniel Martin Klotz, Ruming Yang, Melanie Brux, Frank Buchholz, Hani Harb, Theresa Link, Pauline Wimberger, Mirko Theis, Jan Dominik Kuhlmann

**Affiliations:** 1 Department of Gynecology and Obstetrics, Medical Faculty and University Hospital Carl Gustav Carus, Technische Universität Dresden, Dresden, Germany; 2 National Center for Tumor Diseases/University Cancer Center (NCT/UCC): German Cancer Research Center (DKFZ), Heidelberg, Germany, Faculty of Medicine and University Hospital Carl Gustav Carus, Technische Universität Dresden, Helmholtz-Zentrum Dresden-Rossendorf (HZDR), Dresden, Germany; 3 German Cancer Research Center (DKFZ), Heidelberg and German Cancer Consortium (DKTK) Partner Site, Dresden, Germany; 4 Medical Systems Biology, Medical Faculty Carl Gustav Carus, Technische Universität Dresden, Dresden, Germany; 5 Institute for Medical Microbiology and Virology, Medical Faculty and University Hospital Carl Gustav Carus, Technische Universität Dresden, Dresden, Germany

**Keywords:** Ovarian cancer, Drug development

## Abstract

PARP-inhibitors (PARPi) are an integral part of ovarian cancer treatment. However, overcoming acquired PARPi resistance or increasing the benefit of PARPi in patients without homologous recombination deficiency (HRD) remains an unmet clinical need. We sought to identify genetic modulators of PARPi response, guiding pharmacological PARPi sensitization. CRISPR-Cas9 mediated loss-of-function screen with a focused sgRNA library revealed that DNA-demethylases JMJD1B/JMJD1C, targetable by the small inhibitor methylstat, promote PARPi resistance. Methylstat synergistically interacted with olaparib, and (re-)sensitized ovarian cancer cells to PARPi treatment, surpassing the efficacy of common demethylase inhibitors. Genetic knockout of JMJD1B and/or JMJD1C phenocopied the effect of methylstat in an additive manner. Validation studies revealed methylstat to be a universal PARPi-sensitizing drug, effective, regardless of PARPi resistance status or *BRCA1* mutational background. Methylstat modulated clonal cancer dynamics by mitigating positive selection of PARPi-resistant or *BRCA1*-proficient cells under olaparib treatment. Using a model of PARPi-induced cellular toxicity, we showed that methylstat impairs cellular DNA repair, indicated by an increased susceptibility of ovarian cancer cells to olaparib-induced DNA double strand breaks after methylstat exposure. This study proposes the histone demethylase inhibitor methylstat as an epigenetic drug for overcoming PARPi-resistance or for increasing efficacy of PARPi beyond HRD in ovarian cancer patients.

## Introduction

Ovarian cancer is the leading cause of death among gynecological malignancies [1]. As early disease is mostly symptom-free, about 75% of ovarian cancer patients are diagnosed at advanced stages. Standard treatment of advanced ovarian cancer consists of radical debulking surgery, aiming at macroscopic complete tumor resection and platinum/paclitaxel-based chemotherapy, in addition to maintenance treatment with antiangiogenic bevacizumab [2–4]. In patients with homologous recombination (HR) deficiency (HRD), defined by either genomic instability and/or a pathogenic breast cancer 1/2, early onset (*BRCA1/2*) mutation, a combination of bevacizumab and the poly ADP ribose polymerase inhibitor (PARPi) olaparib has been approved as maintenance therapy after response to first-line platinum-based chemotherapy [5]. Moreover, part of standard treatment is (i) olaparib and bevacizumab in HRD positive patients (ii) olaparib monotherapy in patients with *BRCA1/2* mutation or (iii) niraparib independently of HRD-status [6, 7]. In the platinum-sensitive recurrent setting, the PARPis olaparib, niraparib and rucaparib can be used as maintenance therapy, regardless of HRD in high grade serious ovarian cancer [8–11]. Nevertheless, the majority of patients with recurrent ovarian cancer develop PARPi resistance, resulting in poor overall prognosis. Therefore, uncovering the genetic basis of PARPi response is of high clinical interest, in order to guide the design of innovative targeted therapy approaches for ovarian cancer patients.

We have previously established an in vitro model of PARPi-resistant ovarian cancer by long-term exposure of *BRCA1*-deficient UWB1.289 ovarian cancer cell lines to olaparib [12]. The resulting derivative cell-line (Olres-UWB1.289) exhibited an EMT-like phenotype and was characterized by a broad spectrum of cross-resistance toward other clinically relevant PARPis and chemotherapeutic drugs. Using a dual-fluorescence clonal competition assay, we demonstrated that Olres-UWB1.289 cells mirror typical dynamics of therapy resistance, shaped by their strong positive selection under olaparib treatment, their abilility to outcompete PARPi-sensitive cells in co-culture, and their “fitness penalty” under drug-free conditions [12].

Loss-of-function genetic screening by CRISPR/Cas9 has been proven as powerful tool for exploring the genetic basis of various disease-related processes [13, 14], including PARPi response in cancer [15–19]. However, the repertoire of genes, involved in PARPi-resistant ovarian cancer, has incompletely been studied and its clinical translation remains elusive. Therefore, using our previously established model system of acquired PARPi-resistance [12], we aimed at identifying genetic determinants of PARPi response. To this end, we performed a CRISPR-Cas9 mediated loss-of-function screening with a focused sgRNA library, targeting 1.572 human genes, including kinases, cell surface proteins and epigenetic regulators. Identified gene candidates were finally used to design and experimentally validate an optimal pharmacological strategy for PARPi sensitization in ovarian cancer.

## Material and Methods

### Cell Culture

All cell lines were maintained in a humidified incubator with 5% CO_2_ at 37 °C. Detailed information on the composition of culture media, is summarized in Supplementary Table 1. The cell lines were tested regularly for mycoplasm by a commercial in-house service.

### Generation of PARPi-resistant cell lines

PARPi-resistant Olres-UWB1.289 and Olres-UWB1.289+BRCA1 were generated as described previously [12]. Briefly, parental UWB1.289 or UWB1.289+BRCA1 cells had been cultured under continuous exposure to incrementally ascending olaparib concentrations for 11 months, beginning from 10 nM. Final olaparib maintenance level for PARPi-resistant derivative cell lines was adjusted, according to the inherent olaparib sensitivity of the parental cell lines, resulting in 10 µM or 60 µM, respectively [12]. PARPi-resistant Igrov-1, KB1P and KB1P+BRCA1 cells were generated in analogy to our previous model, with final maintenance levels of 60 µM, 4 µM or 60 µM olaparib, respectively.

### Drugs

Olaparib (AZD2281; #S1060, PARP1) [20], 1-Napthyl-PP1 (#S2642, target: ABL1) [21, 22], Imatinib (#S2475, target: ABL1) [23–27], Dasatinib (#S1021, target: ABL1) [25, 28–30], Sodium orthovanadate (#S2000, target: ATP1A3) [31, 32], Remodelin hydrobromide (#S7641, target: NAT10) [33], Entrectinib (#S7998, target: PAN3-NTRK2 fusion, TRIM24-NTRK2 fusion) [34, 35], Larotrectinib (#S5860, target: PAN3-NTRK2 fusion, TRIM24-NTRK2 fusion) [34, 36], Dorsomorphin dihydrochloride (#S7306, target: AMPK and its subunit PRKAB2) [37], Selumetinib (#S1008, target: STK35-BRAF fusion, TRIM24-BRAF fusion) [38], Stattic (#S7024; target: STAT3, upstream of STK35) [39, 40], PFI-90 (#S9882; JMJD1B) [41], GSK-J1 (#S7581, KDM6A, KDM6B) [42–44], IOX-1 (#S7234, target: JMJD1A, KDM4C, KDM6B, KDM2A, KDM4E, KDM5C, PHD2, ALKBH5) [45, 46], GSK-LSD1 2HCl (#S7574, target: LSD1) [47, 48] were purchased from Selleckchem (Munich, Germany). Methylstat (#SML0343; selective inhibitor of Jumonji C domain-containing histone demethylases, including JMJD1B, JMJD1C) [49] was purchased from Sigma Aldrich and Metformin (#317240-5 G, target: SP1) [50] from EMD Millipore.

### CRISPR-Cas9 library design

The sgRNA library was designed to cover six protein classes, i.e., kinases, cell surface proteins [51], nuclear receptors, epigenetic factors, transcription factors and uncharacterized genes (Supplementary Table 2). Each gene was targeted with 3-12 different sgRNAs, selected to specifically bind to either the first exon, an early splicing site or the functional domain of the protein. All sgRNAs were chosen to fulfill the criteria defined by Doench et al. [52]. The complete library consisted of 10,722 sgRNAs targeting 1572 genes. 636 sgRNAs were included to target 45 essential and 47 non-essential control genes [53] (Supplementary Table 2). Oligonucleotides with sgRNA sequences were ordered as arrayed synthesis from CustomArray Inc. and PCR amplified (primer, forward: 5′-GATATTGCAACGTCTCACACC-3′, reverse: 5′-GTCGCGTACGTCTCGAAAC-3′). The resulting PCR product was cloned into the lentiviral vector pL.CRISPR.EFS.tRFP (Addgene; #57819) using the Esp3I restriction sites. The vector contained a modified tracr sequence (5′-GTTTAAGAGCTATGCTGGAAACAGCATAGCAAGTTTAAATAAGGCTAGTCCGTTATCAACTTGAAAAAGTGGCACCGAGTCGGTGCTTTTTTT-3′), as described previously [54].

### Lentiviral CRISPR-Cas9 loss-of-function screen

Lentivirus production was adapted from our previously described protocol [12, 55]. HEK293T cells were transfected with a three-plasmid split-genome packaging system. Per viral transduction of 0.5 × 10^6^ Olres-UWB1.289 target cells, 12 µg of the pL.CRISPR.EFS.tRFP transfer vector (Addgene, 57818) DNA, encoding for *Streptococcus pyogenes* Cas9 and a sgRNA from the library, respectively, was mixed with 12 µg psPAX2 DNA (Addgene, 12260), 6 µg pMD2.G DNA (Addgene, 12259) and 62 µl of 2 M CaCl_2_ in a final volume of 500 µl. Subsequently 500 µl of 2x HBS phosphate buffer was dropwise added to the mixture and incubated for 10 min at room temperature (RT). The 1 ml transfection mixture was then added to 50% confluent HEK293T cells, seeded the day before into a 10 mm dish. Cells were incubated for 16 h (37 °C and 5% CO_2_) and the medium was changed to remove remaining transfection reagent. Lentiviral-supernatants were collected 36 h post-transfection and for each infection, 3 ml supernatant containing 4 mg/ml polybrene was immediately used to infect Olres-UWB1.289 cells [12], seeded the day before in 10 mm dishes to reach around 50% confluency at infection. Infected cells were incubated for 24 h, followed by a medium change to remove virus particles. Six days after infection, transduction efficiency was determined by flow cytometric tRFP analysis, followed by fluorescence-activated cell sorting (FACS) of tRFP-positive cells using a BD FACSAria™ Fusion cell sorter. According to titration experiments, viral supernatant from HEK293T cells was 10-fold diluted with culture medium to result in 30% transduction efficacy. In order to enable a representative projection of the CRISPR-Cas9 library on target cells, a total of 2.25 × 10^7^ Olres-UWB1.289 cells were transduced, followed by sorting of 2.4 million tRFP-positive cells and a 19-day neutral expansion period. For recording baseline library composition, genomic DNA (gDNA) was extracted from five million cells using the QiAmp DNA blood kit (Qiagen, 51104). The remaining population was maintained in culture. Finally, two parallel sets of library-transduced tRFP-positive cells were seeded (5 million each, T75 flask format) for i) growth under drug-free conditions and ii) growth under 4 µM olaparib selection pressure. After eight days, gDNA was extracted from both conditions.

Isolated gDNA samples were used to amplify sgRNA sequences by two rounds of PCR, with the second-round primers containing adaptors for Illumina sequencing (PCR 1, forward 5′-GTAATAATTTCTTGGGTAGTTTGCA-3′, reverse 5′-ATTGTGGATGAATACTGCCATTTG-3′; PCR 2, forward 5′-ACACTCTTTCCCTACACGACGCTCTTCCGATCTGGCTTTATATATCTTGTGGAAAGG-3′, reverse 5′- GTGACTGGAGTTCAGACGTGTGCTCTTCCGATCTCAAGTTGATAACGGACTAGCC-3′). After targeted PCR amplification, the samples were indexed for NGS sequencing in a successive PCR enrichment followed by purification and capillary electrophoresis (Fragment Analyzer, Agilent). The resulting libraries were sequenced with single-end reads on a NextSeq 500. The sequence reads were mapped to sgRNA sequences using PatMaN [56], a rapid short sequence aligner. As a set of query patterns, we used sgRNA sequences flanked by 5′-GACGAAACACCG-3′ and 5′-GTTTAAGAGCTA-3′ on the termini, respectively, and allowed two mismatches during the alignment step. For each read, the best matching gRNA sequence was picked. In case of ties, the read was discarded as ambiguous. For each sample, counts of reads, mapped to each sgRNA from the library, were calculated.

### Generation of CRISPR/Cas9 knockout cell lines

For targeted gene knockout, sgRNAs against the respective gene of interest were designed (Supplementary Table 4). As control, a sgRNA targeting a gene desert site in the genome, referred to as safe target (STC3)-control or a non-targeting sgRNA with no homologies to the human genome (nt-control) was used. For lentiviral transduction, the same basic protocol was used as described above. A total of 0.4 × 10^6^ Olres-UWB1.289 were transduced with 0.7-fold diluted viral supernatant, resulting in transduction efficacies below 80% (range 29.4–76.2%) in order to avoid off-target effects, due to excessive integration events in the target cells. After a culture period of two weeks, genomic DNA (gDNA) was isolated from the transfected cells using the QiAmp DNA Blood Mini Kit (Qiagen, 51104). Subsequently, the gRNA binding region was amplified by PCR and subjected to Sanger sequencing. The indel rates were analyzed using the Inference of CRISPR Edits (ICE) Analysis Tool (Performance Analysis, ICE Analysis, 2019, V3.0, Synthego). In case of a polyclonal knockout efficiency <70%, cell lines were subjected to single cell sorting and monoclonal expansion.

### Photometric Cell Viability Assay

Cell viability assays were performed as described previously [12, 57]. Briefly, cells were seeded on 6-well plates and cultured in standard medium for 24 h to allow adherence. Subsequently, drug treatment was performed for 6 days. On day 7, cells were fixed with 0.1% methanol and stained with 0.1% crystal violet solution. For viability readouts, the cell-bound violet dye fraction was dissolved by 10% acetic acid and photometrically quantified using the Tecan Reader (Infinite M200, Männedorf, Switzerland, Software Magellan Version 7.2). IC_50_ values were determined by non-linear regression using Prism Version 10.0 (GraphPad Software, CA, USA).

### Drug interaction analysis

For drug interaction analysis of the PARPi olaparib with the respective small molecule inhibitor, a series of drug combination treatments at equipotent molar concentrations were performed (IC_6.25_, IC_12.5_, IC_25_, IC_50_ and IC_100_ [58]). Drug interaction was classified, according to photometric cell viability assays and the combination index (CI) method [57, 58]. For each equipotent combination, a CI-value was calculated using CalcuSyn Version 2.11 (Biosoft, Cambridge, UK). A CI-value < 0.9 indicates a synergistic interaction [58]. Synergyfinder2.0, calculating the Bliss and HSA synergy scores, were used as independent reference models for the classification of drug interactions. A score >10 indicates synergy.

### Dual-fluorescence clonal competition assay

The effect of methylstat treatment on the clonal dynamics of PARPi-resistant cells was analyzed, as described previously [12]. Briefly, a mixture of 7,000 cells/well (50% UWB1.289-pWPXL-tdTomato and 50% Olres-UWB1.289-pWPXL-eGFP or 50% UWB+BRCA1-pWPXL-eGFP) were seeded into a black 96-well plate (costar®; Corning Incorporated, 3603) and treated one day later with 10 µM olaparib or the combination of 10 µM olaparib and 3 µM methylstat. eGFP and tdTomato fluorescent cells were counted using the CeligoS Image Cytometer (Nexcelom Bioscience Ltd, Lawrence, MA, USA).

### γH2AX Western Blot analysis

From treated cell lines, 20 µg protein per sample was subjected to a NuPAGE 4–12% Bis-Tris protein gel (Invitrogen by Thermo Fisher Scientific; #NP0321BOX) and transferred onto nitrocellulose (NC) membranes (0.45 µm NC, Thermo Fisher Scientific; #88018). Subsequently, (dissected) NC-membranes were incubated with Anti-ß-Actin (clone AC-74; mouse; Sigma-Aldrich, #A5316; 1:100000) and Anti-phospho-histone H2A.X (Ser139; clone JBW301; mouse; Sigma-Aldrich, #05-636; 1:1000) antibodies. Membranes were incubated for detection with secondary antibodies, raised against mouse (Peroxidase-conjugated AffiniPure Goat anti-Mouse IgG, HRP linked; Jackson ImmunoResearch; #115-035-003). Detection was performed with Amersham™ ECL™ Prime Western Blotting Detection reagent (Cytiva, Freiburg, Germany; #RPN2232). The quantification was performed using the Fiji Software and results were visualized by Prism 10.2.3 (GraphPad Software).

### Statistics

Basic statistical analysis was performed using Prism 10.2.3 (GraphPad Software). Viability assays were analysed by nested t-test and the IC_50_ was determined with nonlinear regression. Drug interaction was assessed using CI-value calculation, Calcusyn 2.11 (Biosoft) and the Bliss/HSA model based on the Synergyfinder2.0 software. For more than two comparisons within selected datasets, the one-way ANOVA test with post-hoc Tukey, Dunnet or Šídák correction was used.

## Results

### CRISPR-Cas9 loss-of-function screen for the identification of genetic determinants of PARPi response

**Fig. 1:**
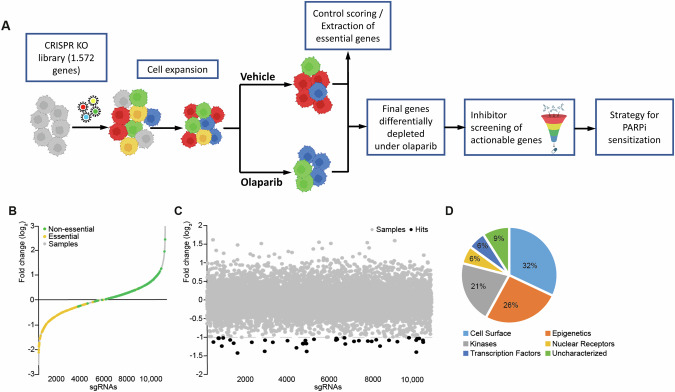
CRISPR-Cas9 loss-of-function screen for the identification genetic determinants of PARPi response. **A** Schematic overview of the CRISPR-Cas9 loss-of-function screen and its translation into a pharmacological strategy for PARPi sensitization. PARPi-resistant Olres-UWB1.289 cells were transduced with a sgRNA library in order to identify genes, negatively selected under olaparib treatment. Pharmacological inhibitors targeting hit genes were screened for a synergistic interaction with olaparib. **B** sgRNA frequencies (log_2_ of fold change) normalized to baseline after gene knockout and cell expansion. Fold changes of sgRNA are shown in gray and highlighted for essential (yellow) and non-essential (green) control genes. **C** Fold changes in the sgRNA pool composition (log_2_) after treatment with olaparib normalized to the vehicle control. sgRNA frequencies (gray) of depleted genes (dashed line) are shown; hit genes (black, red), JMJD1B/C (red) are highlighted. **D** Protein class distribution of hit genes.

We performed a lentiviral pooled loss-of-function screen in PARPi-resistant Olres-UWB1.289 cells [12] using Cas9 from *S. pyogenes*. The screen was based on a tailor-made sgRNA library targeting 1572 human candidate genes that cover a pre-selection of various protein classes including kinases, epigenetic regulators and cell surface proteins (Supplementary Table 2). As a first step, we confirmed appropriate control scoring of our CRISPR-Cas9 setup. To this end, lentiviral delivery of the sgRNA library to Olres-UWB1.289 was performed. Changes in the composition of the pre-defined control sgRNA sequences (*n* = 636) [53] were determined by deep sequencing after neutral expansion of cells for eight days under drug-free conditions (Fig. 1A). While 38 of 45 (84%) essential control genes for cell homeostasis, were depleted (log_2_ <-0.5; Fig. 1B), 283 of 326 sgRNAs (87%) targeting non-essential control genes were increased or only mildly depleted (log_2_ > -0.5; Fig. 1B). This confirmed technical validity of our CRISPR-Cas9 platform and showed that the library composition is accurately mirroring the expected negative or positive selection effects of control knockout genotypes.

As a second step, we excluded additional target genes from our library, whose sgRNA sequences were depleted under the above-mentioned drug-free growth period (log_2_ < -0.5), as they were essential for cell homeostasis and not specifically involved in PARPi response (Fig. 1B). Lastly, we filtered for a cleaned set of hit genes, whose sgRNA sequences were differentially depleted under seven days of olaparib selection pressure by at least two-fold (Fig. 1B–D, Supplementary Table 3). The majority of hit genes were transcription factors (32%), followed by epigenetic regulators (26%) and kinases (21%; Fig. 1D).

Conclusively, we identified a final set of 34 candidate genes, promoting PARPi resistance in Olres-UWB1.289 cells (Fig. 1C, Supplementary Table 3).

### Translation of the CRISPR-Cas9 screening results into a pharmacological strategy for PARPi sensitization

**Fig. 2:**
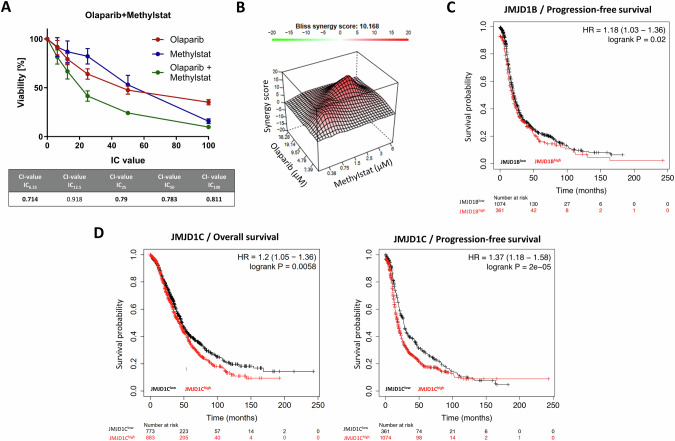
Synergistic interaction of methylstat with the PARPi olaparib in the PARPi-resistant Olres-UWB1.289 model. **A** Drug interaction analysis of methylstat and olaparib according to the combination index (CI) method. Drugs were combined at a broad range of equipotent molar concentrations, according to the indicated IC_100_, IC_50_, IC_25_, IC_12.5_, IC_6,25_. Standard deviation from three independent experiments is indicated by error bars. Resulting CI-values are indicated in the table. A drug interaction with a CI-value < 1.0 is classified as synergistic (bold numbers). **B** Drug interaction analysis of methylstat and olaparib according to Bliss synergy score calculation. A score >10 indicates synergy. Results were derived from three independent biological replicates. Kaplan-Meier analysis showing prognostic relevance of (**C**) JMJD1B and (**D**) JMJD1C according to the publicly available Kaplan-Meier plotter tool. PFS progression-free survival, OS overall survival.

We reasoned that pharmacological targeting of the above-identified genetic determinants of PARPi resistance will result in PARPi (re-)sensitization in Olres-UWB1.289 cells. For 12 of 34 identified candidate genes, commercially available inhibitors on protein level were available (Table 1). For orthogonal validation of the CRISPR-Cas9 screening results, we tested each inhibitor for a potential synergistic interaction with olaparib in Olres-UWB1.289 cells [12], using the combination index (CI) method [58] (Supplementary Fig. 1). Most of the inhibitors, particularly dorsomorphin (targeting *PRKAB2* [37]), imatinib (targeting *ABL1* [23–27]) or larotrectinib (targeting *PAN3*, *TRIM24* [34, 36]), exhibited mostly antagonistic or additive interaction with olaparib, indicated by CI-values > 0.9 (Table 1; Supplementary Fig. 1). Methylstat, a small molecule inhibitor of Jumonji C domain-containing histone demethylases (JMJD) [49], targeting JMJD1B and JMJD1C proteins, was the drug with the most consistent synergistic interaction with olaparib across the total dosage range (CI = 0.71–0.81; Table 1; Fig. 2A), suggesting it as candidate drug for PARPi sensitization in ovarian cancer. A synergistic interaction of methylstat and olaparib was confirmed by independent reference models (Bliss/HSA synergy score; Fig. 2B, Supplementary Fig. 2). According to public databases (TCGA), we inferred that high expression of either JMJD1B or JMJD1C indicates poor prognosis in ovarian cancer patients, corroborating their pro-tumorigenic function in ovarian cancer (Fig. 2C, D).

**Table 1:** Pharmacological inhibitors screened for synergistic interactions with olaparib.

Inhibitor	Target	CI-value IC_6.25_	CI-value IC_12.5_	CI-value IC_25_	CI-value IC_50_	CI-value IC_100_	Percentage synergistic CI-values	Synergy score Bliss	Synergy score HSA
Methylstat	**JMJD1B/JMJD1C**	**0.714**	0.928	**0.779**	**0.783**	**0.811**	73	**10.168**	**18.31**
Entrectinib	**PAN3**-NTRK2 fusion, **TRIM24**-NTRK2 fusion	0.982	**0.814**	1.107	0.942	**0.684**	60	**10.05**	**18.2**
Dasatinib	**ABL1**	1.396	1.134	**0.82**	**0.576**	**0.696**	60	3.27	**13.17**
Metformin	**SP1**	**0.874**	**0.814**	**0.755**	1.011	1.485	60	2.62	**16.12**
Selumetinib	**STK35**-BRAF fusion, **TRIM24**-BRAF fusion	0.944	1.073	**0.884**	**0.575**	**0.783**	60	-0.64	**11.03**
1-Naphthyl-PP1	**ABL1**	**0.751**	**0.896**	1.026	1.152	1.106	60	5.46	**18.37**
Remodelin HBr	**NAT10**	0.963	**0.712**	**0.557**	**0.722**	**0.83**	60	1.78	**13.56**
Stattic	STAT3 (upstream of **STK35**)	2.454	1.378	1.028	**0.711**	**0.619**	53	3.66	**11.49**
Sodium orthovanadate	**ATP1A3** (NaK-ATPase)	1.406	1.420	1.062	**0.860**	1.003	33	n.a.	n.a.
Larotrectinib	**PAN3**-NTRK2 fusion, **TRIM24**-NTRK2 fusion	5.139	2.012	1.416	0.975	1.066	20	-4.70	2.07
Imatinib	**ABL1**	1.201	0.988	1.041	1.109	1.401	0	-0.61	6.17
Dorsomorphin	AMPK and its subunit **PRKAB2**	3.681	1.745	1.119	1.139	1.416	0	-4.00	3.24

A drug interaction with a CI-value < 0.9 is classified as synergistic (bold numbers).

Inhibitors were ranked, according to their synergistic interaction with olaparib as determined by the CI-value along series of drug combination treatments at equipotent molar concentrations. A CI-value < 0.9 indicates a synergistic interaction (bold numbers). The percentage of synergistic interactions along the tested dosage ranges were calculated. Drug interaction were independently validated by the Synergyfinder2.0 algorithm, calculating the Bliss and HSA synergy scores. A score >10 indicates synergy (bold numbers).

**Fig. 3:**
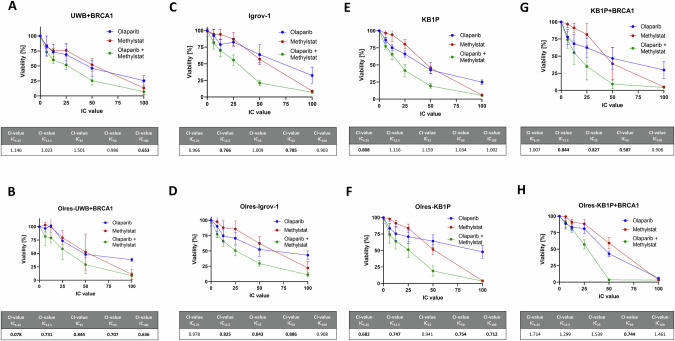
Synergistic interaction of methylstat with the PARPi olaparib in independent in vitro models of PARPi resistance. Drug interaction analysis of methylstat and olaparib according to the combination index (CI) method with the indicated cell lines (**A**–**H**). Drugs were combined at a broad range of equipotent molar concentrations, according to the indicated IC_100_, IC_50_, IC_25_, IC_12.5_, IC_6,25_. Standard deviation from three independent experiments is indicated by error bars. Resulting CI-values are indicated in the table. A drug interaction with a CI-value < 1.0 is classified as synergistic (bold values). Results were derived from three independent biological replicates.

The PARPi sensitizing effect of methylstat could be further validated by independent in vitro models of PARPi-resistance according to human ovarian cancer (Supplementary Fig. 3A; Fig. 3A–D) or murine breast cancer (Supplementary Fig. 3B-C; Fig. 3E–H). In this context, methylstat treatment resulted in a broad spectrum of olaparib sensitization among PARPi-resistant (Olres-UWB+BRCA1, Olres-Igrov-1, Olres-KB1P, Olres-KB1P+BRCA1; Fig. 3B, D, F, H) and PARPi-sensitive (UWB+BRCA1, Igrov-1, KB1P, KB1P+BRCA1; Fig. 3A, C, E, G) cell lines. This effect was independent from the *BRCA1* mutational status of the cells, as we observed a synergistic interaction between methylstat and olaparib among isogenic pairs of *BRCA1*-proficient *vs*. *BRCA1*-deficient cell lines (Fig. 3A, B, E, F, G, H). Interestingly, the most efficient PARPi sensitization effect by methylstat was observed in the cell line with the lowest inherent PARPi response (*BRCA1*-proficient/PARPi-resistant Olres-KB1P cells), already resulting in a complete eradication of cells, when both drugs were combined at the IC_50_ (Fig. 3H).

Taken together, we provide several lines of evidence that pharmacological targeting of the histone demethylases JMJD1B/C by the inhibitor methylstat results in an efficient PARPi sensitization in ovarian cancer cells, independently of the inherent PARPi-resistance status or the *BRCA1* mutational background.

### Effect of JMJD1B/C genetic depletion on PARPi sensitivity

**Fig. 4:**
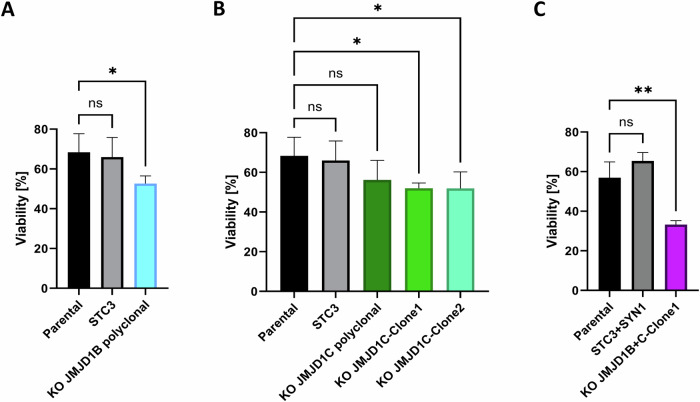
Olaparib sensitivity in Olres-UWB1.289 cells upon genetic depletion of the histone demethylases JMJD1B/C. Viability of monoclonal or polyclonal PARPi-resistant Olres-UWB1.289 knockout cell lines for (**A**) *JMJD1B* (**B**) *JMJD1C* or (**C**) *JMJD1B* *+* *C* compared the control sgRNA-knockout (STC3/SYN1) or parental Olres-UWB1.289 is shown. P-values according to the one-way ANOVA with post-hoc Tukey test are indicated; * padj < 0.05; ** padj < 0.01. Results were derived from three independent biological replicates.

To verify the functional contribution of JMJD1B/C in PARPi response, we performed genetic depletion of the *JMJD1B* gene by CRISPR-Cas9 in PARPi-resistant Olres-UWB1.384 cells. This resulted in a polyclonal derivative cell line with 72% *JMJD1B* knockout efficiency, showing a mild but significantly increased olaparib sensitivity compared to parental cells or the STC3 control knockout (difference 15.77%, padj = 0.04; Fig. 4A, Supplementary Table 5). Upon *JMJD1C* knockout, while olaparib response was not altered at a polyclonal knockout efficiency of 67%, monoclonal expansion resulted in two homozygous *JMJD1C* knockout clones with mildly decreased olaparib sensitivity (difference 16.4%, padj=0.04; 16.5%, padj = 0.03, respectively; Fig. 4B, Supplementary Table 5). Assuming that the histone demethylases JMJD1B/C cooperatively promote PARPi resistance in an additive manner, we subsequently generated homozygous monoclonal *JMJD1B* and *JMJD1C* knockout cells. Interestingly, the double knockout strongly increased olaparib sensitivity (difference 23.69%, padj=0.0039; Fig. 4C, Supplementary Table 5).

Conclusively, genetic depletion of *JMJD1B* and *JMJD1C* phenocopied the PARPi sensitizing effect of methylstat in an additive manner, indicating a central role of these genes in promoting PARPi resistance.

### Methylstat outperforms common demethylase inhibitors in their PARPi sensitization capacity

Using the Igrov-1 model of PARPi-resistant ovarian cancer, we compared the PARPi sensitizing effect of methylstat with commercially available DNA histone demethylases, including JMJD-specific inhibitors GSK-J1 (JMJD3, KDM6A) [42–44] and PFI-90 (JMJD1B) [41] and GSK-LSD1 [47, 48], a non-JMJD demethylase LSD1 inhibitor. Moreover, we tested the pan-demethylase inhibitor IOX1, targeting JMJD and non-JMJD demethylases [45, 46].

**Fig. 5:**
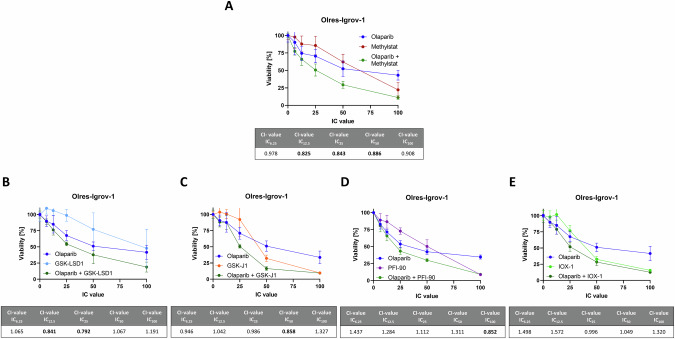
Comparison of the PARPi sensitizing effect of methylstat with that of common histone demethylase inhibitors. Drug interaction analysis of the indicated histone demethylase inhibitors with olaparib according to the combination index (CI) method with the indicated cell lines (**A**–**E**). Drugs were combined at a broad range of equipotent molar concentrations, according to the indicated inhibitory concentration (IC)_100_, IC_50_, IC_25_, IC_12.5_, IC_6,25_. Standard deviation from three independent experiments is indicated by error bars. Resulting CI-values are indicated in the table. A drug interaction with a CI-value < 0.9 is classified as synergistic (bold numbers). Results were derived from three independent biological replicates.

Among the total concentration range tested, GSK-LSD1 showed two synergistic interactions at IC_12.5_ and IC_25_, whereas GSK-J1, IOX-1 and PFI-90 showed only additive or a maximum of one synergistic interaction with olaparib. Most of the tested inhibitors showed PARPi sensitizing effect to certain degrees, however, they were clearly outperformed by the effect of methylstat, which provided synergistic interactions at IC_12.5_, IC_25_ and IC_50_ concentration levels (Fig. 5).

We conclude that methylstat is the most efficient PARPi sensitizer among the tested JMJD and non-JMJD histone demethylase inhibitors GSK-LSD1, GSK-J1 and PFI-90, as well as the pan-demethylase inhibitor IOX1.

### Methylstat modulates clonal dynamics of PARPi-resistant or BRCA1-proficient ovarian cancer cells under olaparib selection pressure

**Fig. 6:**
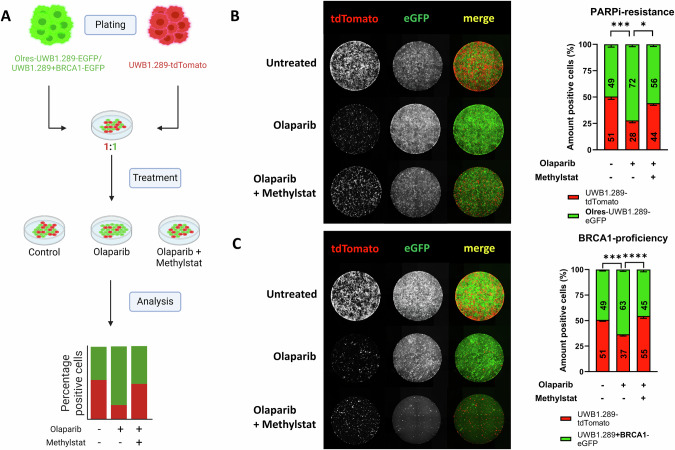
Effect of methylstat treatment on the clonal dynamics of PARPi-resistant Olres-UWB1.289 cells. **A** Schematic overview of a dual-fluorescence clonal competition model. Part of the scheme has been created with Biorender.com. Fluorescent microscopy images of (**B**) PARPi-resistant Olres-UWB1.289-EGFP *vs*. PARPi sensitive UWB1.289-tdTomato and (**C**) *BRCA1*-deficient UWB1.289-tdTomato vs. *BRCA1*-proficient UWB1.289+BRCA1-EGFP cell lines, co-cultured in the presence of drug-free medium, olaparib or methylstat+olaparib. P-values according to the one-way ANOVA with post-hoc Tukey HSD test are indicated; * padj < 0.05; ** padj < 0.01; *** padj < 0.001. Results were derived from three independent biological replicates.

Taking advantage of our previously established dual-fluorescence clonal competition assay [12], we were interested in the effect of methylstat on the clonal dynamics of PARPi-resistant cells (Fig. 6A). Using lentiviral transduction, we color-coded PARPi-resistant (Olres-UWB1.289) with a green (eGFP) and parental PARPi-sensitive cells (UWB1.289) with a red (tdTomato) fluorescent marker protein [12]. After 14 days in co-culture under olaparib selection pressure, we observed a strong positive selection of PARPi-resistant compared to PARPi-sensitive cells (Fig. 6B; padj=0.0003). According to the previously described synergistic interaction between olaparib and methylstat, combined treatment with both drugs resulted in a strong reduction of overall cell numbers. Interestingly, methylstat mitigated the positive selection of PARPi-resistant cells, indicated by a stabilization of the ratio between resistant and sensitive cells towards a 50/50 proportion (Fig. 6B; padj=0.014).

We subsequently investigated the effect of methylstat in a further co-culture model without acquired PARPi resistance, including parental *BRCA1*-deficient cells (UWB1.289+BRCA1) and their *BRCA1*-proficient isogenic counterparts (UWB1.289+BRCA1). Due to their inherently lower PARPi sensitivity, olaparib monotreatment resulted in a strong positive selection of *BRCA1*-proficient cells (Fig. 6C; padj=0.0007), which was mitigated by combined treatment with olaparib and methylstat (Fig. 6C; padj<0.0001).

Taken together, we demonstrate that methylstat mitigates positive selection of either PARPi-resistant or *BRCA1*-proficient ovarian cancer cells under olaparib treatment.

### Methylstat increases susceptibility towards olaparib induced DNA double strand break formation

**Fig. 7:**
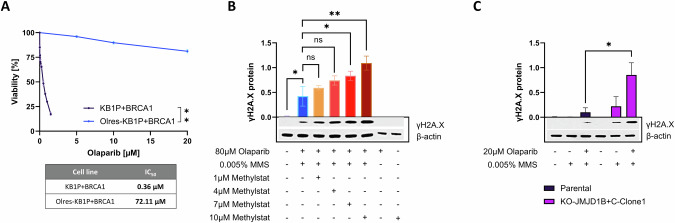
Methylstat increases susceptibility of DNA double strand break formation in olaparib treated Olres-KP1B + BRCA1 cells. **A** Olaparib dose-response curves according to 6-day photometric viability assays of BRCA1-proficient KB1P murine breast cancer cells in comparison to their respective isogenic derivative cell lines with experimentally acquired PARPi-resistance. IC_50_ values are indicated according to non-linear regression analysis of normalized drug response. p-value levels were calculated according to nested t-test of dose-response curves. γH2AX response of (**B**) Olres-KB1P+BRCA1 cells and (**C**) paired Olres-UWB1.289/Olres-UWB1.289-*JMJD1B*/J*MJD1C* double knockout cells, treated with the indicated drug combinations. Representative Western Blot images including densitometric analysis from three independent biological replicates are shown. P-value levels were calculated according to one-way ANOVA with post-hoc Dunnet (**B**) or Šídák (**C**) correction. *p < 0.05; **p < 0.01; ***p < 0.001; ****p < 0.0001.

It is widely accepted that the increased formation of DNA double strand breaks (DSBs) is one of the presumed mechanisms of PARPi associated toxicity in cancer cells with HRD [59]. Using phosphorylated histone H2AX (yH2AX) as a marker for DSBs [59], we were interested in the effect of methylstat on olaparib induced DSBs. For this purpose, we used Olres-KB1P-G3 + BRCA1 cells with the lowest inherent PARPi response among our in vitro models, due to their *BRCA1*-proficient background and their experimentally acquired PARPi resistance (IC_50_ = 72 µM; Fig. 7A). Consistent with this phenotype, short-term treatment of 80 µM olaparib did not yet result in any detectable γ-H2AX signal. However, olaparib, combined with a low dose of the alkylating agent methyl methanesulfonate (MMS; 0.005%), lead to a moderate γ-H2AX induction, providing a cellular model of PARPi-associated DSBs (Fig. 7B). Interestingly, while methylstat alone did not have any effect on γ-H2AX, it potentiated olaparib/MMS induced γ-H2AX induction in a dose-dependent manner (Fig. 7A, B).

We moreover showed that Olres-UWB1.289 *JMJD1B*/*JMJD1C* double knockout cells were characterized by an inherently higher γ-H2AX response to combined olaparib/MMS treatment, compared to parental Olres-UWB1.289 cells. This indicates that the methylstat effect on DNA-damage response is likely mediated by the JMJD1B/JMJD1C demethylases (Fig. 7C).

Conclusively, we demonstrate that methylstat increases susceptibility to olaparib-induced DSBs in PARPi-resistant ovarian cancer cells with a *BRCA1*-proficient background.

## Discussion

Understanding the cellular and genetic determinants of PARPi response is one of the key priorities for clinical translation. Consequently, there has been considerable interest for investigating the hallmarks of PARPi mechanisms via CRISPR screens in cells with diverse genetic backgrounds [15–19]. In our study, we leveraged a well-characterized model of human PARPi-resistant ovarian cancer [12] and conducted a CRISPR-Cas9 knockout screening to systematically explore genetic determinants of PARPi response. Our screen has been conducted on a restricted set of genes, primarily chosen by their categorization into specific protein classes. However, we identified various hit genes for downstream analysis. Although our control scoring showed a high reliability, we cannot exclude that some PARPi-related genes remained undetected, because Cas9-mediated gene editing exhibits notable variability, contingent upon specific genomic target sites [52]. Additionally, our screen has demonstrated a relatively limited dynamic range, preventing a phenotypic ranking of these hit genes from the available dataset. Nevertheless, we have identified and orthogonally validated the histone demethylases JMJD1B/C as negative genetic regulators for PARPi response. Regarding clinical translation, we showed that JMJD1B/C targeting methylstat efficiently (re-)sensitized PARPi-resistant Olres-UWB1.289 cells to olaparib.

Exploiting collateral sensitivities of resistant cancer cells can be a useful strategy for designing targeted therapy approaches. In a preclinical study on breast cancer cells with acquired CDK4/6 inhibitor (CDK4/6i) resistance, authors discovered a collateral sensitivity towards the Wee-1 inhibitor adavosertib, which specifically depleted only CDK4/6i-resistant but not CDK4/6i-sensitive cells in a co-culture setting [60]. Consequently, combined treatment with CDK4/6i and adavosertib allowed tumor control by simultaneously targeting sensitive and resistant breast cancer cells [60]. In contrast, we show that methylstat alone had cytotoxic effects on both, PARPi-sensitive and PARPi-resistant ovarian cancer cells, while additionally potentiating the effect of olaparib among PARPi-resistant, PARPi-sensitive and *BRCA1*-proficient cell lines. This indicates that methylstat targets basic mechanisms of PARPi response, that are shared between PARPi-resistant and sensitive cells. Therefore, the effect of methylstat is not exclusively based on a selective vulnerability of PARPi-resistant cells.

Consistent with this finding, we demonstrated that methylstat increased susceptibility of DSB formation in ovarian cancer cells under olaparib treatment. Moreover, PARPi sensitization was particularly strong in cell lines with a *BRCA1*-proficient background, suggesting that methylstat promotes a pharmacologically induced state of HRD, also referred to as “BRCAness”, which results in synthetic lethality under olaparib treatment [61]. This hypothesis is partially corroborated by previous work, demonstrating that JMJD1C orchestrates chromatin response to DSBs and selectively promotes RAP80-BRCA1-mediated HR [62]. However, in this study, JMJD1C was shown to restrict the formation of RAD51 repair foci, and JMJD1C depletion caused resistance to ionizing radiation or PARPis. This is in contrast to our results could be attributed to the use of another cell line in this study (U2OS sarcoma cells), thus complicating a direct comparison between studies. Interestingly, the JMJD1C-mediated response to DSBs was supposed to result from JMJD1C-mediated demethylation of the non-histone protein MDC1 and not by the canonical histone DNA demethylation, suggesting that JMJD demethylases may control HR by a spectrum of non-canonical and yet unexplored target proteins [62–64]. Nevertheless, methylstat also sensitized ovarian cancer cell lines with a *BRCA1*-deficient background. JMJD1C/B are epigenetic regulators, that control a variety of biological functions in normal tissues and tumors [63] and their inhibition by methylstat likely interferes with additional cancer associated pathways and DNA-repair routes, awaiting further investigation. In acute myeloid leukemia, methylstat was reported to induce synergistic interactions with doxorubicin or the proteasome inhibitor bortezomib [65, 66]. This indicates divers and context-dependent phenotypes upon pharmacological inhibition of JMJD histone demethylases in cancer cells.

In the platinum-sensitive recurrent setting, the PARPis olaparib, niraparib and rucaparib can be clinically used as maintenance therapy in ovarian cancer patients, regardless of the HRD status. However, patients without *BRCA1/2* mutations still have inferior response to PARPi, compared to patients with *BRCA1/2* mutations [8–11]. Therefore, methylstat could be used for enhancing therapeutic efficacy of PARPi beyond *BRCA1/2*-deficiency. Furthermore, combined PARPi and methylstat treatment could be an ideal strategy for overcoming PARPi resistance in ovarian cancer. Lastly, we propose that methylstat could increase the efficacy of PARPi rechallenge in patients, which so far provided only modest PFS benefits [67].

We showed that high JMJD1B or JMJD1C expression indicates poor prognosis in ovarian cancer patients, which has also been reported for breast cancer patients [68] and therefore corroborates a pro-tumorigenic role of these epigenetic regulators. Moreover, combined genetic depletion of *JMJD1C/B* phenocopied the PARPi sensitizing effect of methylstat in an additive manner. Methylstat is a JMJD histone demethylases inhibitor, which is not entirely specific for JMJD1B/C [49]. It is possible that combined depletion of *JMJD1B/C* still does not phenocopy the full extent of the methylstat effect, since JMJD histone demethylases built a complex functional network in cancer [63]. Further members of this family, not targeted by our sgRNA library but inhibited by methylstat, may additionally contribute to PARPi resistance. Moreover, a contribution of histone demethylases beyond the JMJD family to PARPi resistance seems likely, because we showed that GSK-LSD1, targeting lysine specific demethylase 1 (LSD1), also provided a mild PARPi sensitizing effect. Pharmacological LSD1 inhibition has already been reported to confer anti-tumor activity in pre-clinical studies, to sensitize for chemotherapy, and to enhance anti-tumor immune response [69, 70]. Moreover, it has been investigated in a clinical trial on acute myeloid leukemia or lung cancer (NCT02177812, NCT02034123). Nevertheless, methylstat provided the most efficient PARPi sensitizing effect among all tested inhibitors of JMJD family and non-JMJD family members, potentially qualifying this drug for a preferred clinical use in ovarian cancer.

## Conclusion

We propose that pharmacological targeting of the demethylase JMJD1B/C by the inhibitor methylstat sensitizes ovarian cancer cells to PARPi. Our findings are of high clinical-translational relevance, as they (i) provide a novel concept for increasing efficacy of PARPi inhibitors in ovarian cancer beyond HRD and (ii) pave the way for an alternative treatment option in patients with acquired PARPi resistance.

## Supplementary information

Supplementary Figure and Table Legends

Supplementary Figures

Supplementary Table 1

Supplementary Table 2

Supplementary Table 3

Supplementary Table 4

Supplementary Table 5

## Data Availability

All relevant data have been included into the manuscript. Raw data can be made available upon reasonable request to the last authors.

## References

[1] Siegel R, Ma J, Zou Z, Jemal A. Cancer statistics, 2014. CA: a cancer J clinicians. 2014;64:9–29. 10.3322/caac.21208 24399786

[2] du Bois A, Quinn M, Thigpen T, Vermorken J, Avall-Lundqvist E, Bookman M, et al. 2004 consensus statements on the management of ovarian cancer: final document of the 3rd International Gynecologic Cancer Intergroup Ovarian Cancer Consensus Conference (GCIG OCCC 2004). Ann Oncol. 2005;16:viii7–viii12. 16239238 10.1093/annonc/mdi961

[3] Karam A, Ledermann JA, Kim JW, Sehouli J, Lu K, Gourley C, et al. Fifth ovarian cancer consensus conference of the gynecologic cancer InterGroup: first-line interventions. Ann Oncol. 2017;28:711–7. 28327917 10.1093/annonc/mdx011

[4] Stuart GC, Kitchener H, Bacon M, duBois A, Friedlander M, Ledermann J, et al. 2010 Gynecologic Cancer InterGroup (GCIG) consensus statement on clinical trials in ovarian cancer: report from the Fourth Ovarian Cancer Consensus Conference. Int J Gynecol Cancer. 2011;21:750–5. 21543936 10.1097/IGC.0b013e31821b2568

[5] Ray-Coquard I, Pautier P, Pignata S, Perol D, Gonzalez-Martin A, Berger R, et al. Olaparib plus bevacizumab as first-line maintenance in ovarian cancer. N Engl J Med. 2019;381:2416–28. 31851799 10.1056/NEJMoa1911361

[6] Moore K, Colombo N, Scambia G, Kim BG, Oaknin A, Friedlander M, et al. Maintenance olaparib in patients with newly diagnosed advanced ovarian cancer. N Engl J Med. 2018;379:2495–505. 30345884 10.1056/NEJMoa1810858

[7] Gonzalez-Martin A, Pothuri B, Vergote I, DePont Christensen R, Graybill W, Mirza MR, et al. Niraparib in patients with newly diagnosed advanced ovarian cancer. N. Engl J Med. 2019;381:2391–402. 31562799 10.1056/NEJMoa1910962

[8] Ledermann J, Harter P, Gourley C. Olaparib maintenance therapy in patients with platinum-sensitive relapsed serous ovarian cancer: a preplanned retrospective analysis of outcomes by BRCA status in a randomised phase 2 trial. Lancet Oncol. 2015;16:e158. Correction to Lancet Oncol 2014; 15: 856 24882434 10.1016/S1470-2045(14)70228-1

[9] Pujade-Lauraine E, Ledermann JA, Selle F, Gebski V, Penson RT, Oza AM, et al. Olaparib tablets as maintenance therapy in patients with platinum-sensitive, relapsed ovarian cancer and a BRCA1/2 mutation (SOLO2/ENGOT-Ov21): a double-blind, randomised, placebo-controlled, phase 3 trial. Lancet Oncol. 2017;18:1274–84. 28754483 10.1016/S1470-2045(17)30469-2

[10] Mirza MR, Monk BJ, Herrstedt J, Oza AM, Mahner S, Redondo A, et al. Niraparib maintenance therapy in platinum-sensitive, recurrent ovarian cancer. N. Engl J Med. 2016;375:2154–64. 27717299 10.1056/NEJMoa1611310

[11] Coleman RL, Oza AM, Lorusso D, Aghajanian C, Oaknin A, Dean A, et al. Rucaparib maintenance treatment for recurrent ovarian carcinoma after response to platinum therapy (ARIEL3): a randomised, double-blind, placebo-controlled, phase 3 trial. Lancet. 2017;390:1949–61. 28916367 10.1016/S0140-6736(17)32440-6 PMC5901715

[12] Klotz DM, Schwarz FM, Dubrovska A, Schuster K, Theis M, Kruger A, et al. Establishment and molecular characterization of an in vitro model for PARPi-resistant ovarian cancer. Cancers (Basel). 2023;15:3774. 37568590 10.3390/cancers15153774 PMC10417418

[13] Awwad SW, Serrano-Benitez A, Thomas JC, Gupta V, Jackson SP. Revolutionizing DNA repair research and cancer therapy with CRISPR-Cas screens. Nat Rev Mol Cell Biol. 2023;24:477–94. 36781955 10.1038/s41580-022-00571-x

[14] Sreevalsan S, Doring M, Paszkowski-Rogacz M, Brux M, Blanck C, Meyer M, et al. MLLT6 maintains PD-L1 expression and mediates tumor immune resistance. EMBO Rep. 2020;21:e50155. 33063451 10.15252/embr.202050155 PMC7726806

[15] Pettitt SJ, Krastev DB, Brandsma I, Drean A, Song F, Aleksandrov R, et al. Genome-wide and high-density CRISPR-Cas9 screens identify point mutations in PARP1 causing PARP inhibitor resistance. Nat Commun. 2018;9:1849. 29748565 10.1038/s41467-018-03917-2 PMC5945626

[16] Ipsen MB, Sorensen EMG, Thomsen EA, Weiss S, Haldrup J, Dalby A, et al. A genome-wide CRISPR-Cas9 knockout screen identifies novel PARP inhibitor resistance genes in prostate cancer. Oncogene. 2022;41:4271–81. 35933519 10.1038/s41388-022-02427-2

[17] Tsujino T, Takai T, Hinohara K, Gui F, Tsutsumi T, Bai X, et al. CRISPR screens reveal genetic determinants of PARP inhibitor sensitivity and resistance in prostate cancer. Nat Commun. 2023;14:252. 36650183 10.1038/s41467-023-35880-y PMC9845315

[18] Clements KE, Schleicher EM, Thakar T, Hale A, Dhoonmoon A, Tolman NJ, et al. Identification of regulators of poly-ADP-ribose polymerase inhibitor response through complementary CRISPR knockout and activation screens. Nat Commun. 2020;11:6118. 33257658 10.1038/s41467-020-19961-w PMC7704667

[19] Fang P, De Souza C, Minn K, Chien J. Genome-scale CRISPR knockout screen identifies TIGAR as a modifier of PARP inhibitor sensitivity. Commun Biol. 2019;2:335. 31508509 10.1038/s42003-019-0580-6 PMC6733792

[20] Evers B, Drost R, Schut E, de Bruin M, van der Burg E, Derksen PW, et al. Selective inhibition of BRCA2-deficient mammary tumor cell growth by AZD2281 and cisplatin. Clin Cancer Res. 2008;14:3916–25. 18559613 10.1158/1078-0432.CCR-07-4953

[21] Tandon M, Johnson J, Li Z, Xu S, Wipf P, Wang QJ. New pyrazolopyrimidine inhibitors of protein kinase d as potent anticancer agents for prostate cancer cells. PLoS One. 2013;8:e75601. 24086585 10.1371/journal.pone.0075601 PMC3781056

[22] Bishop AC, Ubersax JA, Petsch DT, Matheos DP, Gray NS, Blethrow J, et al. A chemical switch for inhibitor-sensitive alleles of any protein kinase. Nature. 2000;407:395–401. 11014197 10.1038/35030148

[23] Druker BJ, Sawyers CL, Kantarjian H, Resta DJ, Reese SF, Ford JM, et al. Activity of a specific inhibitor of the BCR-ABL tyrosine kinase in the blast crisis of chronic myeloid leukemia and acute lymphoblastic leukemia with the Philadelphia chromosome. N. Engl J Med. 2001;344:1038–42. 11287973 10.1056/NEJM200104053441402

[24] Schultz KR, Carroll A, Heerema NA, Bowman WP, Aledo A, Slayton WB, et al. Long-term follow-up of imatinib in pediatric Philadelphia chromosome-positive acute lymphoblastic leukemia: Children’s Oncology Group study AALL0031. Leukemia. 2014;28:1467–71. 24441288 10.1038/leu.2014.30 PMC4282929

[25] Ottmann O, Dombret H, Martinelli G, Simonsson B, Guilhot F, Larson RA, et al. Dasatinib induces rapid hematologic and cytogenetic responses in adult patients with Philadelphia chromosome positive acute lymphoblastic leukemia with resistance or intolerance to imatinib: interim results of a phase 2 study. Blood. 2007;110:2309–15. 17496201 10.1182/blood-2007-02-073528

[26] Soverini S, Colarossi S, Gnani A, Rosti G, Castagnetti F, Poerio A, et al. Contribution of ABL kinase domain mutations to imatinib resistance in different subsets of Philadelphia-positive patients: by the GIMEMA Working Party on Chronic Myeloid Leukemia. Clin Cancer Res. 2006;12:7374–9. 17189410 10.1158/1078-0432.CCR-06-1516

[27] Cortes J, Jabbour E, Kantarjian H, Yin CC, Shan J, O’Brien S, et al. Dynamics of BCR-ABL kinase domain mutations in chronic myeloid leukemia after sequential treatment with multiple tyrosine kinase inhibitors. Blood. 2007;110:4005–11. 17785585 10.1182/blood-2007-03-080838

[28] Cortes JE, Saglio G, Kantarjian HM, Baccarani M, Mayer J, Boque C, et al. Final 5-year study results of DASISION: the dasatinib versus imatinib study in treatment-naive chronic myeloid leukemia patients trial. J Clin Oncol. 2016;34:2333–40. 27217448 10.1200/JCO.2015.64.8899 PMC5118045

[29] Kantarjian H, Shah NP, Hochhaus A, Cortes J, Shah S, Ayala M, et al. Dasatinib versus imatinib in newly diagnosed chronic-phase chronic myeloid leukemia. N. Engl J Med. 2010;362:2260–70. 20525995 10.1056/NEJMoa1002315

[30] Soverini S, Colarossi S, Gnani A, Castagnetti F, Rosti G, Bosi C, et al. Resistance to dasatinib in Philadelphia-positive leukemia patients and the presence or the selection of mutations at residues 315 and 317 in the BCR-ABL kinase domain. Haematologica. 2007;92:401–4. 17339191 10.3324/haematol.10822

[31] Cantley LC Jr., Josephson L, Warner R, Yanagisawa M, Lechene C, Guidotti G. Vanadate is a potent (Na,K)-ATPase inhibitor found in ATP derived from muscle. J Biol Chem. 1977;252:7421–3. 144127

[32] Yang-Feng TL, Schneider JW, Lindgren V, Shull MM, Benz EJ Jr., Lingrel JB, et al. Chromosomal localization of human Na+, K+-ATPase alpha- and beta-subunit genes. Genomics. 1988;2:128–38. 2842249 10.1016/0888-7543(88)90094-8

[33] Larrieu D, Britton S, Demir M, Rodriguez R, Jackson SP. Chemical inhibition of NAT10 corrects defects of laminopathic cells. Science. 2014;344:527–32. 24786082 10.1126/science.1252651 PMC4246063

[34] Drilon A, Siena S, Ou SI, Patel M, Ahn MJ, Lee J, et al. Safety and antitumor activity of the multitargeted pan-TRK, ROS1, and ALK inhibitor entrectinib: combined results from two phase I trials (ALKA-372-001 and STARTRK-1). Cancer Discov. 2017;7:400–9. 28183697 10.1158/2159-8290.CD-16-1237 PMC5380583

[35] Cho BC, Chiu CH, Massarelli E, Buchschacher GL, Goto K, Overbeck TR, et al. Updated efficacy and safety of entrectinib in NTRK fusion-positive non-small cell lung cancer. Lung Cancer. 2024;188:107442. 38171156 10.1016/j.lungcan.2023.107442

[36] Doebele RC, Davis LE, Vaishnavi A, Le AT, Estrada-Bernal A, Keysar S, et al. An oncogenic NTRK fusion in a patient with soft-tissue sarcoma with response to the tropomyosin-related kinase inhibitor LOXO-101. Cancer Discov. 2015;5:1049–57. 26216294 10.1158/2159-8290.CD-15-0443 PMC4635026

[37] Lu Y, Akinwumi BC, Shao Z, Anderson HD. Ligand activation of cannabinoid receptors attenuates hypertrophy of neonatal rat cardiomyocytes. J Cardiovasc Pharm. 2014;64:420–30. 10.1097/FJC.0000000000000134 24979612

[38] Fangusaro J, Onar-Thomas A, Young Poussaint T, Wu S, Ligon AH, Lindeman N, et al. Selumetinib in paediatric patients with BRAF-aberrant or neurofibromatosis type 1-associated recurrent, refractory, or progressive low-grade glioma: a multicentre, phase 2 trial. Lancet Oncol. 2019;20:1011–22. 31151904 10.1016/S1470-2045(19)30277-3 PMC6628202

[39] Schust J, Sperl B, Hollis A, Mayer TU, Berg T. Stattic: a small-molecule inhibitor of STAT3 activation and dimerization. Chem Biol. 2006;13:1235–42. 17114005 10.1016/j.chembiol.2006.09.018

[40] Wu Z, Liu J, Hu S, Zhu Y, Li S. Serine/threonine kinase 35, a target gene of STAT3, regulates the proliferation and apoptosis of osteosarcoma cells. Cell Physiol Biochem. 2018;45:808–18. 29414823 10.1159/000487172

[41] Kim YY, Gryder BE, Sinniah R, Peach ML, Shern JF, Abdelmaksoud A, et al. KDM3B inhibitors disrupt the oncogenic activity of PAX3-FOXO1 in fusion-positive rhabdomyosarcoma. Nat Commun. 2024;15:1703. 38402212 10.1038/s41467-024-45902-y PMC10894237

[42] Kruidenier L, Chung CW, Cheng Z, Liddle J, Che K, Joberty G, et al. A selective jumonji H3K27 demethylase inhibitor modulates the proinflammatory macrophage response. Nature. 2012;488:404–8. 22842901 10.1038/nature11262 PMC4691848

[43] Yang D, Okamura H, Teramachi J, Haneji T. Histone demethylase Utx regulates differentiation and mineralization in osteoblasts. J Cell Biochem. 2015;116:2628–36. 25920016 10.1002/jcb.25210

[44] Wang JJ, Wang X, Xian YE, Chen ZQ, Sun YP, Fu YW, et al. The JMJD3 histone demethylase inhibitor GSK-J1 ameliorates lipopolysaccharide-induced inflammation in a mastitis model. J Biol Chem. 2022;298:102017. 35526564 10.1016/j.jbc.2022.102017 PMC9168612

[45] Schiller R, Scozzafava G, Tumber A, Wickens JR, Bush JT, Rai G, et al. A cell-permeable ester derivative of the JmjC histone demethylase inhibitor IOX1. ChemMedChem. 2014;9:566–71. 24504543 10.1002/cmdc.201300428 PMC4503230

[46] Cheng P, Han H, Chen F, Cheng L, Ma C, Huang H, et al. Amelioration of acute myocardial infarction injury through targeted ferritin nanocages loaded with an ALKBH5 inhibitor. Acta Biomater. 2022;140:481–91. 34879293 10.1016/j.actbio.2021.11.041

[47] Wang Z, Long QY, Chen L, Fan JD, Wang ZN, Li LY, et al. Inhibition of H3K4 demethylation induces autophagy in cancer cell lines. Biochim Biophys Acta Mol Cell Res. 2017;1864:2428–37. 28800922 10.1016/j.bbamcr.2017.08.005

[48] Zhang W, Ruan X, Li Y, Zhi J, Hu L, Hou X, et al. KDM1A promotes thyroid cancer progression and maintains stemness through the Wnt/beta-catenin signaling pathway. Theranostics. 2022;12:1500–17. 35198054 10.7150/thno.66142 PMC8825597

[49] Luo X, Liu Y, Kubicek S, Myllyharju J, Tumber A, Ng S, et al. A selective inhibitor and probe of the cellular functions of Jumonji C domain-containing histone demethylases. J Am Chem Soc. 2011;133:9451–6. 21585201 10.1021/ja201597b PMC3133600

[50] Nair V, Pathi S, Jutooru I, Sreevalsan S, Basha R, Abdelrahim M, et al. Metformin inhibits pancreatic cancer cell and tumor growth and downregulates Sp transcription factors. Carcinogenesis. 2013;34:2870–9. 23803693 10.1093/carcin/bgt231 PMC3845888

[51] Bausch-Fluck D, Hofmann A, Bock T, Frei AP, Cerciello F, Jacobs A, et al. A mass spectrometric-derived cell surface protein atlas. PLoS ONE. 2015;10:e0121314. 25894527 10.1371/journal.pone.0121314 PMC4404347

[52] Doench JG, Fusi N, Sullender M, Hegde M, Vaimberg EW, Donovan KF, et al. Optimized sgRNA design to maximize activity and minimize off-target effects of CRISPR-Cas9. Nat Biotechnol. 2016;34:184–91. 26780180 10.1038/nbt.3437 PMC4744125

[53] Evers B, Jastrzebski K, Heijmans JP, Grernrum W, Beijersbergen RL, Bernards R. CRISPR knockout screening outperforms shRNA and CRISPRi in identifying essential genes. Nat Biotechnol. 2016;34:631–3. 27111720 10.1038/nbt.3536

[54] Chen B, Gilbert LA, Cimini BA, Schnitzbauer J, Zhang W, Li GW, et al. Dynamic imaging of genomic loci in living human cells by an optimized CRISPR/Cas system. Cell. 2013;155:1479–91. 24360272 10.1016/j.cell.2013.12.001 PMC3918502

[55] Liffers ST, Munding JB, Vogt M, Kuhlmann JD, Verdoodt B, Nambiar S, et al. MicroRNA-148a is down-regulated in human pancreatic ductal adenocarcinomas and regulates cell survival by targeting CDC25B. Lab Invest. 2011;91:1472–9. 21709669 10.1038/labinvest.2011.99

[56] Prufer K, Stenzel U, Dannemann M, Green RE, Lachmann M, Kelso J. PatMaN: rapid alignment of short sequences to large databases. Bioinformatics. 2008;24:1530–1. 18467344 10.1093/bioinformatics/btn223 PMC2718670

[57] Wichmann C, Klotz DM, Zeiler HJ, Hilger RA, Grutzmann K, Kruger A, et al. The effect of the triazene compound CT913 on ovarian cancer cells in vitro and its synergistic interaction with the PARP-inhibitor olaparib. Gynecol Oncol. 2020;159:850–9. 32980128 10.1016/j.ygyno.2020.09.018

[58] Reynolds CP, Maurer BJ. Evaluating response to antineoplastic drug combinations in tissue culture models. Methods Mol Med. 2005;110:173–83. 15901935 10.1385/1-59259-869-2:173

[59] Bryant HE, Schultz N, Thomas HD, Parker KM, Flower D, Lopez E, et al. Specific killing of BRCA2-deficient tumours with inhibitors of poly(ADP-ribose) polymerase. Nature. 2005;434:913–7. 15829966 10.1038/nature03443

[60] Grolmusz VK, Chen J, Emond R, Cosgrove PA, Pflieger L, Nath A, et al. Exploiting collateral sensitivity controls growth of mixed culture of sensitive and resistant cells and decreases selection for resistant cells in a cell line model. Cancer Cell Int. 2020;20:253. 32565737 10.1186/s12935-020-01337-1 PMC7301982

[61] Abbotts R, Dellomo AJ, Rassool FV. Pharmacologic induction of BRCAness in BRCA-proficient cancers: expanding PARP inhibitor use. Cancers (Basel). 2022;14:2640. 35681619 10.3390/cancers14112640 PMC9179544

[62] Watanabe S, Watanabe K, Akimov V, Bartkova J, Blagoev B, Lukas J, et al. JMJD1C demethylates MDC1 to regulate the RNF8 and BRCA1-mediated chromatin response to DNA breaks. Nat Struct Mol Biol. 2013;20:1425–33. 24240613 10.1038/nsmb.2702

[63] Sui Y, Gu R, Janknecht R. Crucial functions of the JMJD1/KDM3 epigenetic regulators in cancer. Mol Cancer Res. 2021;19:3–13. 32605929 10.1158/1541-7786.MCR-20-0404 PMC7772267

[64] Lu J, Matunis MJ. A mediator methylation mystery: JMJD1C demethylates MDC1 to regulate DNA repair. Nat Struct Mol Biol. 2013;20:1346–8. 24304913 10.1038/nsmb.2729 PMC4131721

[65] Kaci FN, Kiraz Y, Cekdemir D, Baran Y. Synergistic apoptotic effects of bortezomib and methylstat on multiple myeloma cells. Arch Med Res. 2020;51:187–93. 32111493 10.1016/j.arcmed.2020.01.012

[66] Koca D, Hastar N, Engur S, Kiraz Y, Ulu GT, Cekdemir D, et al. Therapeutic potentials of inhibition of Jumonji C domain-containing demethylases in acute myeloid leukemia. Turk J Haematol. 2020;37:5–12. 31833715 10.4274/tjh.galenos.2019.2019.0083 PMC7057756

[67] Pujade-Lauraine E, Selle F, Scambia G, Asselain B, Marme F, Lindemann K, et al. Maintenance olaparib rechallenge in patients with platinum-sensitive relapsed ovarian cancer previously treated with a PARP inhibitor (OReO/ENGOT-ov38): a phase IIIb trial. Ann Oncol. 2023;34:1152–64. 37797734 10.1016/j.annonc.2023.09.3110

[68] Wang L, Chang J, Varghese D, Dellinger M, Kumar S, Best AM, et al. A small molecule modulates Jumonji histone demethylase activity and selectively inhibits cancer growth. Nat Commun. 2013;4:2035. 23792809 10.1038/ncomms3035 PMC3724450

[69] Zhou Z, Van der Jeught K, Fang Y, Yu T, Li Y, Ao Z, et al. An organoid-based screen for epigenetic inhibitors that stimulate antigen presentation and potentiate T-cell-mediated cytotoxicity. Nat Biomed Eng. 2021;5:1320–35. 34725507 10.1038/s41551-021-00805-x PMC8647932

[70] Zhang XY, Hao P, Wang JW, Zhao W, Liu HM, He PX. Inhibition of lysine-specific demethylase 1 enhances the sensitivity of the chemotherapeutic drug doxorubicin in gastric cancer cell. Mol Biol Rep. 2023;50:507–16. 36352181 10.1007/s11033-022-07960-7

