# Supplementary material for: Methylstat sensitizes ovarian cancer cells to PARP-inhibition by targeting the histone demethylases JMJD1B/C
Source: Cancer Gene Ther. 2025 Feb 6;32(3):286–96. doi: 10.1038/s41417-025-00874-z (PMC11946898; doi:10.1038/s41417-025-00874-z)
Supplement: Supplementary file 1 — Supplementary Figure and Table Legends [file 41417_2025_874_MOESM1_ESM.docx]

**Supplementary Figure Legends**

**Supplementary Figure 1:** Drug interaction analysis of methylstat and olaparib according to the combination index (CI) method. Drugs were combined at a broad range of equipotent molar concentrations, according to the indicated IC_100_, IC_50_, IC_25_, IC_12.5_, IC_6.25_. Standard deviation from three independent experiments is indicated by error bars. Resulting CI-values and a ranking according to CI-values are indicated in the Table 1. Results were derived from three independent biological replicates.

**Supplementary Figure 2:** Drug interaction analysis of methylstat and olaparib according to HSA synergy score. A score >10 indicates synergy.

**Supplementary Figure 3: Basic characterization of in vitro models for PARPi-resistance.** The figure shows olaparib dose-response curves according to 6-day photometric viability assays of (**A**) Igrov-1 human ovarian cancer cells (**B**) *BRCA1*-deficient KB1P murine breast cancer (**C**) BRCA1-proficient KB1P murine breast cancer cells in comparison to their respective isogenic derivative cell lines with experimentally acquired PARPi-resistance. PARPi resistance was acquired by chronic exposure to incrementally ascending concentrations of olaparib. IC_50_ values are indicated according to non-linear regression analysis of normalized drug response; p-value levels were calculated according to nested t-test of dose-response curves; **p < 0.01, **** p < 0.0001. Results were derived from three independent biological replicates.

**Supplementary Table Legends**

**Supplementary Table 1: Cell lines and cell culture medium composition.** Cell line names and the components of the respective cell culture medium with supplier and catalog number are indicated.

**Supplementary Table 2: gRNA library composition including protein classes and target gene names.** Genomic target site and efficiency scores for all sgRNAs and the number of potential off-target sites (mismatches, mm 1-4 mm) are given as indicated.

**Supplementary Table 3: Results of Olaparib screen.** Counts from Next Generation Sequencing of the gRNA library before (baseline) and after culturing with and without olaparib treatment as shown.

**Sublementary Table 4: gRNA and primer sequences.** A) Sequences of gRNAs used for gene knockouts. B) Oligonucleotide sequences used for PCR amplifications.

**Supplementary table 5: Genotyping.** Genotyping data of CRISPR knockout cells including gRNA sequences, genomic target sites and results from Inference of CRISPR Edits (IDE) analysis as indicated.
