# Supplementary material for: Methylstat sensitizes ovarian cancer cells to PARP-inhibition by targeting the histone demethylases JMJD1B/C
Source: Cancer Gene Ther. 2025 Feb 6;32(3):286–96. doi: 10.1038/s41417-025-00874-z (PMC11946898; doi:10.1038/s41417-025-00874-z)
Supplement: Supplementary file 2 — Supplementary Figures [file 41417_2025_874_MOESM2_ESM.pptx]

## Slide 1
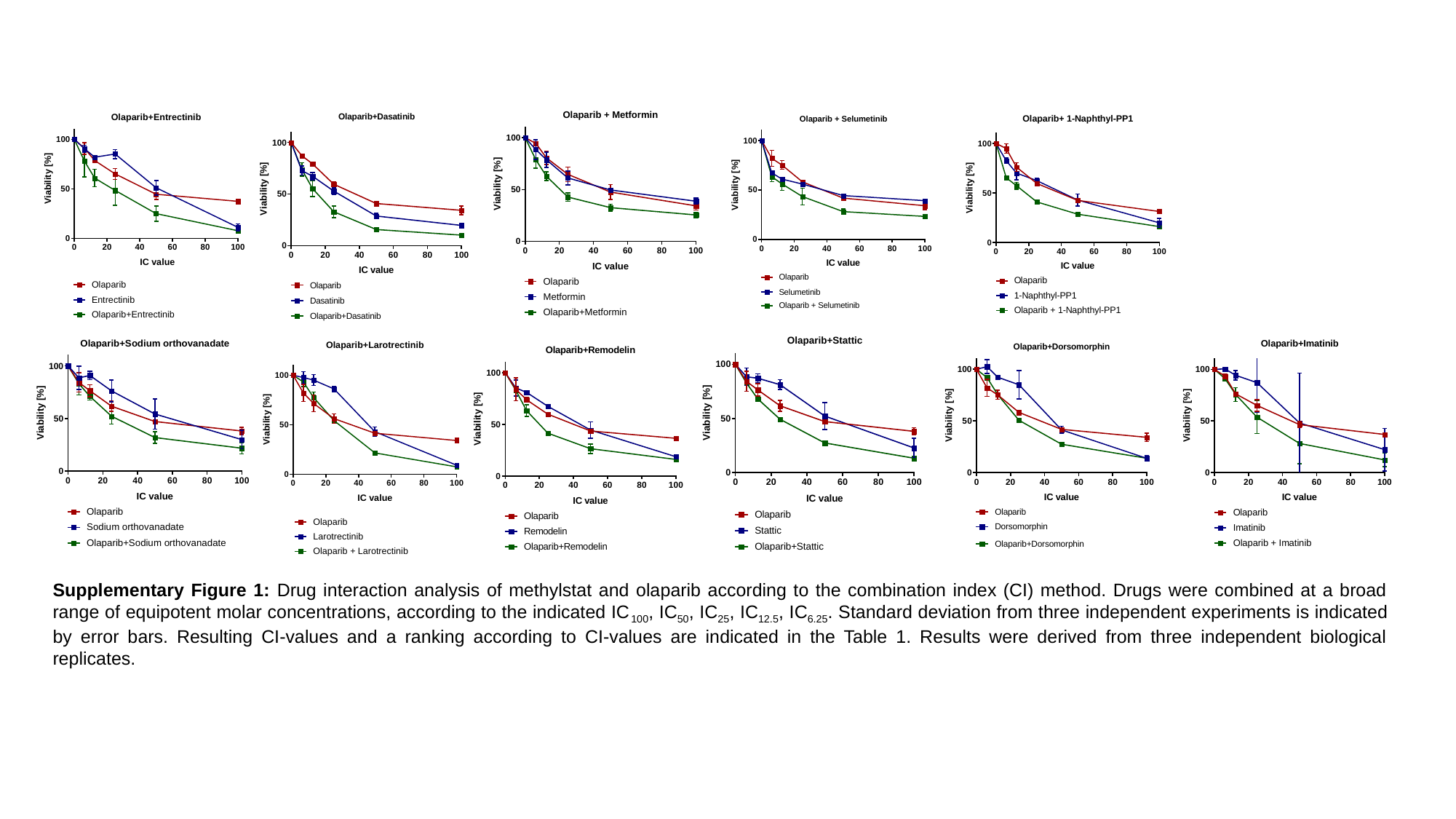

Supplementary Figure 1: Drug interaction analysis of methylstat and olaparib according to the combination index (CI) method. Drugs were combined at a broad range of equipotent molar concentrations, according to the indicated IC100, IC50, IC25, IC12.5, IC6.25. Standard deviation from three independent experiments is indicated by error bars. Resulting CI-values and a ranking according to CI-values are indicated in the Table 1. Results were derived from three independent biological replicates.

## Slide 2
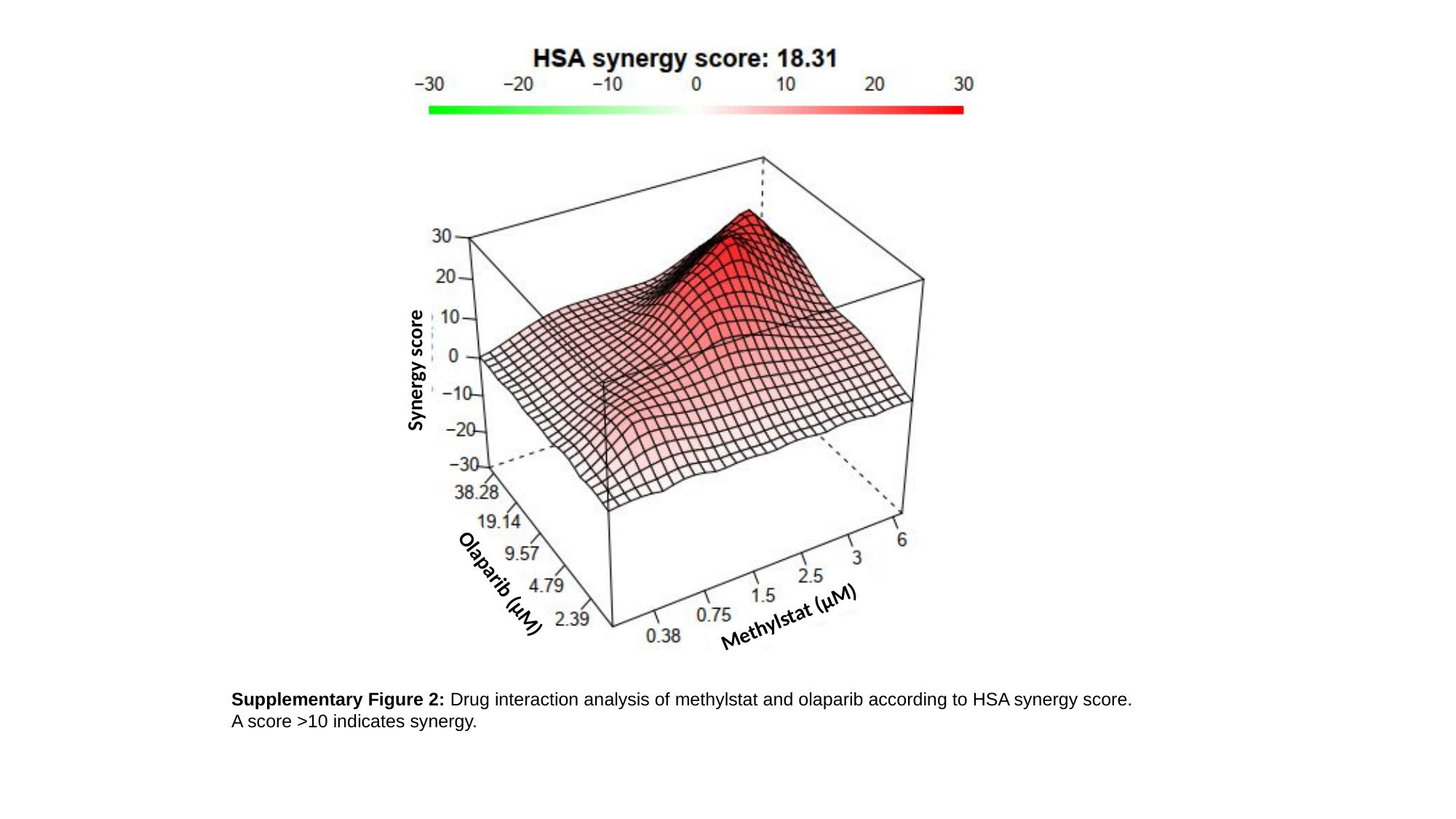

Synergy score
Olaparib (µM)
Methylstat (µM)
Supplementary Figure 2: Drug interaction analysis of methylstat and olaparib according to HSA synergy score. A score >10 indicates synergy.

## Slide 3
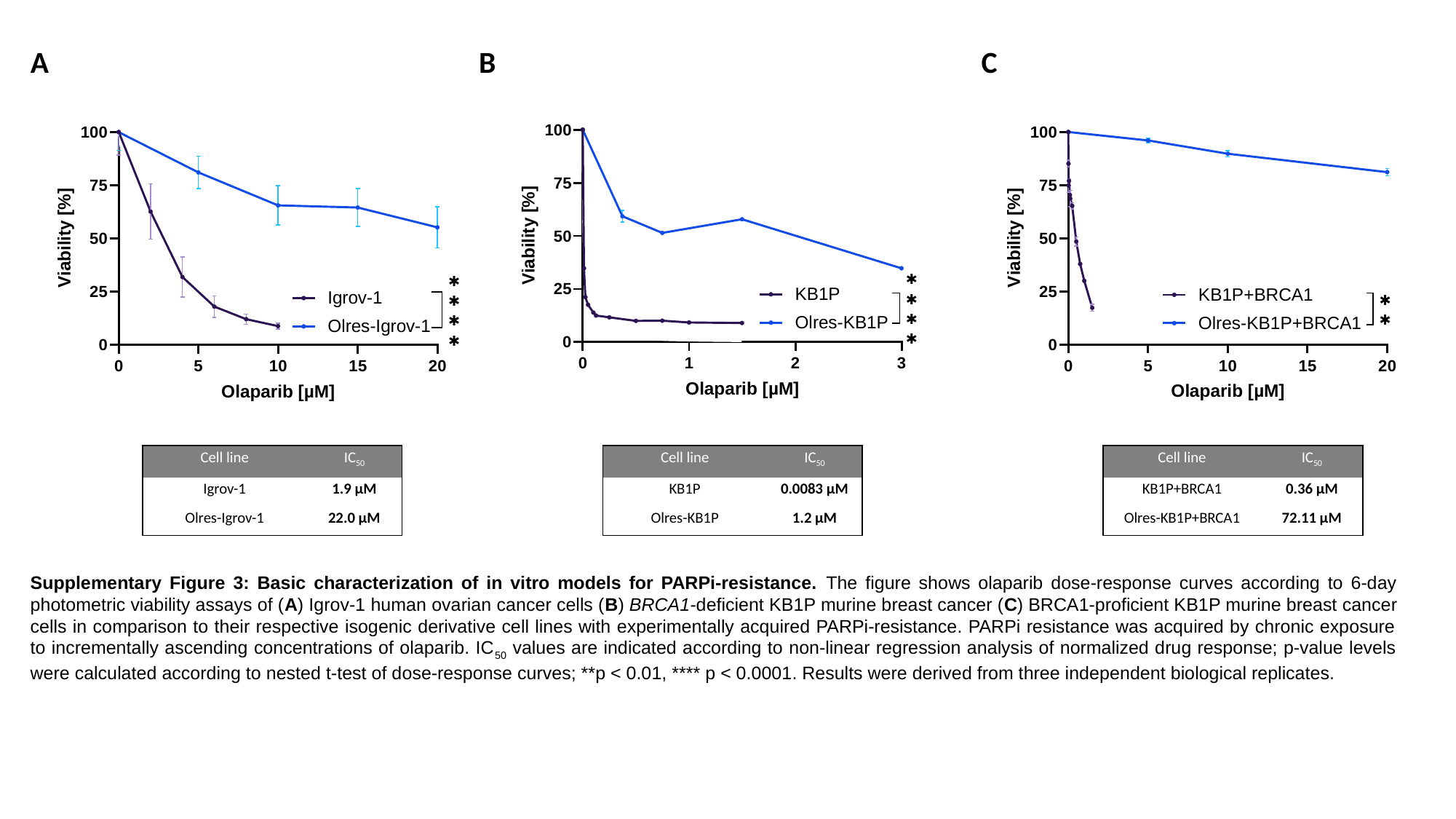

A
B
C
| Cell line | IC50 |
| --- | --- |
| Igrov-1 | 1.9 µM |
| Olres-Igrov-1 | 22.0 µM |
| Cell line | IC50 |
| --- | --- |
| KB1P | 0.0083 µM |
| Olres-KB1P | 1.2 µM |
| Cell line | IC50 |
| --- | --- |
| KB1P+BRCA1 | 0.36 µM |
| Olres-KB1P+BRCA1 | 72.11 µM |
Supplementary Figure 3: Basic characterization of in vitro models for PARPi-resistance. The figure shows olaparib dose-response curves according to 6-day photometric viability assays of (A) Igrov-1 human ovarian cancer cells (B) BRCA1-deficient KB1P murine breast cancer (C) BRCA1-proficient KB1P murine breast cancer cells in comparison to their respective isogenic derivative cell lines with experimentally acquired PARPi-resistance. PARPi resistance was acquired by chronic exposure to incrementally ascending concentrations of olaparib. IC50 values are indicated according to non-linear regression analysis of normalized drug response; p-value levels were calculated according to nested t-test of dose-response curves; **p < 0.01, **** p < 0.0001. Results were derived from three independent biological replicates.
